# Enhancing Biological Control of *Drosophila suzukii*: Efficacy of *Trichopria drosophilae* Releases and Interactions with a Native Parasitoid, *Pachycrepoideus vindemiae*

**DOI:** 10.3390/insects16070715

**Published:** 2025-07-11

**Authors:** Nuray Baser, Charbel Matar, Luca Rossini, Abir Ibn Amor, Dragana Šunjka, Dragana Bošković, Stefania Gualano, Franco Santoro

**Affiliations:** 1 International Centre for Advanced Mediterranean Agronomic Studies of Bari (CIHEAM Bari), 70010 Valenzano, BA, Italy; charbel12matar12@gmail.com (C.M.); abirtunisie@gmail.com (A.I.A.); gualano@iamb.it (S.G.); fsantoro@iamb.it (F.S.); 2 Service d’Automatique et d’Analyse des Systèmes, Universitè Libre de Bruxelles, 1050 Brussels, Belgium; luca.rossini@ulb.be; 3 Department for Environmental and Plant Protection, Faculty of Agriculture, University of Novi Sad, 21000 Novi Sad, Serbia; dragana.sunjka@polj.edu.rs (D.Š.); dragana.boskovic@polj.edu.rs (D.B.)

**Keywords:** spotted wing drosophila, pupal parasitoids, augmentative release, parasitism rate, sustainable pest management, insect interactions, invasive species control, cherry orchard, natural enemies

## Abstract

**Simple Summary:**

*Drosophila suzukii* is an invasive fruit fly that lays its eggs into mature fruits, causing relevant economic damage in soft fruit production. The high number of generations per year and the peak of infestations close to harvest makes control of this pest challenging. Current regulations on pesticide use are increasingly pushing the scientific community toward alternative, low-impact solutions, particularly those involving the use of natural enemies. Accordingly, evaluating the efficacy of entomopathogenic fungi and bacteria, as well as insect predators and parasitoids, through laboratory and field experiments is a pivotal area of research. As *D. suzukii* is an invasive pest worldwide, the evaluation of local and generalist natural enemies depends on the pool of endemic species present in each infested area. This study focuses on the effectiveness of *Trichopria drosophilae* as a biocontrol agent of *D. suzukii* and its potential interaction with *Pachycrepoideus vindemiae*, a naturally occurring parasitoid of *D. suzukii*, in soft fruit orchards in Apulia (Southern Italy). This research focuses on three main questions: (i) Is the population level of parasitoids high enough to reduce insect pest infestations? (ii) Can two prevalent parasitoid species coexist without excessive competition? (iii) How do their interactions affect biological control outcomes? These questions, in our specific case, can be addressed through field trials evaluating the adaptation of *T. drosophilae* and its interaction with *P. vindemiae* in controlling *D. suzukii*, and through laboratory experiments assessing the parasitisation efficiency of *T. drosophilae*, *P. vindemiae*, and their combined effect. The answers to these questions can substantially contribute to improving biological control strategies, highlighting the role of native parasitoids and their interactions in managing an invasive pest species of economic relevance.

**Abstract:**

The spotted wing drosophila, *Drosophila suzukii* is an injurious polyphagous pest threatening worldwide soft fruit production. Its high adaptability to new colonized environments, short life cycle, and wide host range are supporting its rapid spread. The most common techniques to reduce its significant economic damage are based on multiple insecticides applications per season, even prior to the harvest, which reduces agroecosystem biodiversity and affects human and animal health. Environmental concerns and regulatory restrictions on insecticide use are driving the need for studies on alternative biological control strategies. This study aimed to assess the effect of *T. drosphilae* in controlling *D. suzukii* infestations and its interaction with *P. vindemiae*, a secondary parasitoid naturally present in Apulia (South Italy). Field experiments were carried out in organic cherry orchards in Gioia del Colle (Bari, Italy) to test the efficacy and adaptability of *T. drosphilae* following weekly releases of artificially reared individuals. Additionally, the interaction between *P. vindemiae* and *T. drosphilae* was studied under laboratory conditions. Results from field experiments showed that *D. suzukii* populations were significantly lower when both parasitoids were present. However, *T. drosophilae* was less prone to adaptation, so its presence and parasitism were limited to the post-release period. Laboratory experiments, instead, confirmed the high reduction of *D. suzukii* populations when both parasitoids are present. However, the co-existence of the two parasitoids resulted in a reduced parasitism rate and offspring production, notably for *T. drosophilae*. This competitive disadvantage may explain its poor establishment in field conditions. These findings suggest that the field release of the two natural enemies should be carried out with reference to their natural population abundance to not generate competition effects.

## 1. Introduction

*Drosophila suzukii*, commonly known as the spotted wing drosophila (SWD), is widely recognized as one of the most damaging invasive pests of soft and stone fruit crops on a global scale. Its rapid expansion is driven by a combination of biological and ecological traits, including strong dispersal capability, a wide host range, and remarkable adaptability to different climatic conditions. These features, in synergy with the intensification of global trade, have supported its establishment in various areas worldwide and made effective management increasingly complex [1,2,3].

Recent studies have further highlighted the ecological impact and invasion dynamics of SWD, underlying the urgent need for innovative and sustainable biological control approaches [1,2,4,5]. The capability of *D. suzukii* to exploit both cultivated and wild host plants allows its development over the seasons, as spontaneous vegetation act as reservoirs for reinfestation and overwintering shelters [6]. Accordingly, pest control often relies on frequent pesticide applications, which increase production costs and raise serious concerns to environmental and human health [7].

In Italy, *D. suzukii* was first detected in 2009 in the Trentino region, where it rapidly became a major issue in berry orchards, causing economic losses estimated at three million euros by 2011 [8,9]. After the first outbreak, the species subsequently spread across the Italian peninsula, reaching the southern region of Apulia in 2013; since then, significant damages have been recorded in grape, pomegranate, and other fruit crops [10,11]. To date, *D. suzukii* still remains one of the most widespread and destructive pests in fruit production systems worldwide.

To counter its persistent pressure while supporting sustainable agriculture, there has been a gradual shift toward more environmentally conscious and sustainable pest management strategies. Pesticide-based control strategies, in fact, are further complicated by several factors, including the high risk of resistance phenomena, the higher susceptibility of the fruits during the harvest period [12,13,14,15,16], and the negative effect on pollinators, which are fundamental to ensure production for some crops, and on other natural enemies. Over the years, several alternatives have been explored to overtake pesticide usage, such as agronomic practices that remove potential secondary hosts (e.g., wild hosts or fruit leftovers after the harvest), mass trapping, netting, and insect sterilization [13,15,17]. In addition to the promising efficacy of these practices, biological control based on the use of natural enemies has been identified as a valuable component of integrated pest management programs to adapt to local agroecological conditions [18].

Recent advances in pest biological control have emphasized the role of entomopathogenic organisms, such as nematodes, bacteria, fungi, and parasitoids, that target the larval and pupal stages of *D. suzukii*. The entomopathogenic fungi *Beauveria bassiana* and *Metarhizium anisopliae* have shown promising results in reducing the larval and adult populations of *D. suzukii*; under laboratory conditions, mortality rates reached values of 80–90% [19,20]. In addition to the effectiveness in laboratory trials, their performance under field conditions remains inconsistent, frequently inhibited by environmental factors such as low humidity, high solar radiation, and suboptimal timing of application [21]. These limitations have been further assessed by a field study conducted in Apulia [22], testing an autochthonous strain of *M. anisopliae* in an organic cherry orchard. While the fungus showed significant reductions in *D. suzukii* oviposition, its performance varied over the year depending on microclimatic conditions, especially relative humidity and temperature.

On the other hand, promising results have been obtained with insect parasitoid wasps. *Trichopria drosophilae* and *Pachycrepoideus vindemiae* reduce *D. suzukii* populations by more than 50% in laboratory conditions [23] and in semi-field conditions [24]. *T. drosophilae* is a pupal endoparasitoid that develops inside the puparium of various Drosophilid species, ultimately killing the host. The high level of compatibility with *D. suzukii* elects this natural enemy as one of the most promising agents for biological control [25,26,27,28,29]. Laboratory trials have been preliminarily validated [17,30], demonstrating that *T. drosophilae* can locate and parasitize pupae, even when they are concealed within fruits or leaf litter. *P. vindemiae*, instead, is an idiobiont ectoparasitoid [2] of pupae active on flies belonging to Drosophilidae, Muscidae, Anthomyiidae, Calliphoridae, and Sarcophagidae. Female wasps place eggs in the area between the host pupae and the puparium, supported by their serrated ovipositor [26].

The intentional release of a natural enemy would support the sustainable management of invasive species by creating a new ecological balance in infested areas [31]. However, interactions among parasitoids and predators of *D. suzukii* within the same natural environment may strengthen or weaken their parasitism and their effectiveness as biological controllers. These interactions could be of neutral, additive, antagonistic, or have synergistic effects, positively or negatively affecting the biological control of *D. suzukii* [32,33].

Although *T. drosophilae* and *P. vindemiae* are known for their effect on *D. suzukii* populations, their interactions and combined effects on pest level reduction are still poorly explored, above all in Mediterranean and South European environments. This study aims to extend previous works on this subject by assessing the efficacy and adaptability of *T. drosophilae* as a biological control agent of *D. suzukii* while examining its interaction with existing natural enemies under both field and laboratory conditions. The study has been carried out in Southeast Italy, where the climatic conditions might change the equilibria among the species with respect to the areas where previous studies have been conducted. Accordingly, this study enlarges the overview on the pool of natural enemies of *D. suzukii*, further supporting the formulation of ad hoc biological control programs.

## 2. Materials and Methods

### 2.1. Effect of T. drosophilae and Its Interaction with P. vindemiae on the Control of D. suzukii Under Field Conditions

#### 2.1.1. *D. suzukii* Rearing

*D. suzukii* was first collected in 2012 from naturally infested table grapes grown in the experimental fields of CIHEAM Bari, located in Southern Italy. Since then, a continuous rearing colony has been maintained to ensure the availability of specimens for experimental purposes. The genetic variability and fitness of the colony is ensured by the periodic introduction of wild-type adults emerging from infested fruits (primarily cherries and grapes) collected in Gioia del Colle and Turi (Apulia, south Italy). Insects developed in ventilated Plexiglas cages (50 × 40 × 40 cm), as described in [11], were fed with a cornmeal diet medium prepared by mixing 70 g of corn flour, 10 g of soybean meal, yeast, 15 g of brown sugar cane, and 5 g of agar in 1 L of boiling distilled water. The blend was stirred every 2 min for 30 min during boiling. Vitamin mix (2.5 g) and 99% propionic acid (5 mL) were added to the blend, cooled to 25 °C. The blend was further stored at 4 °C before its usage. Water was supplied every 2–3 days [23]. The cages were kept in growth chambers (FDM-Environment, Rome, Italy) under controlled conditions of 24 ± 2 °C, 62 ± 4% RH, 14:10 light–dark (LD).

#### 2.1.2. Parasitoid Rearing

Continuous rearing of *T. drosophilae* was established in 2015, through a colony of adults provided by the Fondazione Edmund Mach (Trento, Italy). To preserve the genetic variability and the fitness of the population, wild-type parasitoid adults emerging from pupae collected in organic fruit orchards were introduced periodically, after morphological identification. Third-instar larvae and young pupae of *D. suzukii* were used as hosts and placed in 50 × 40 × 40 cm Perspex cages. Host preparation began by placing Petri dishes containing an artificial diet in the continuous *D. suzukii* rearing system (see Section 2.1.1) to allow the adult flies to lay eggs. After 3–4 days in a growth chamber, the eggs developed into third-instar larvae, which were then transferred to dedicated cages and exposed to *T. drosophilae* adults for three days to promote parasitism. The adult parasitoids were fed with a solution of honey and sugar provided by water-soaked cotton. After the exposure period, Petri dishes containing the parasitised pupae were incubated under stable environmental conditions (24 ± 2 °C, 62 ± 4% relative humidity and a 14:10 light–dark cycle) for 30 days, allowing the new generation of adult parasitoids to develop and emerge.

#### 2.1.3. *Trichopria drosophilae* Release

**Figure 1:**
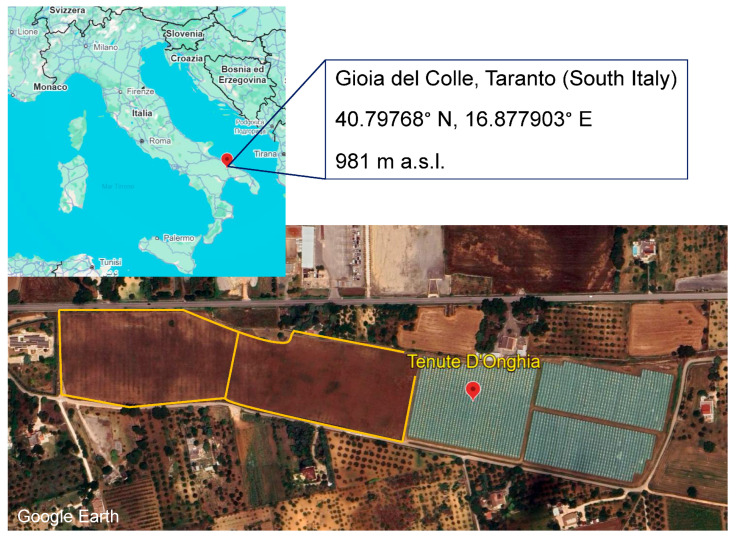
Map showing the release area for parasitoid monitoring in Gioia del Colle, Taranto province, Italy (GPS coordinates: 40.797678°, 16.877903°, elevation 981 m.a.s.l.). The yellow-highlighted fields represent the monitoring plots where parasitoid releases were conducted.

The trial was conducted in an organic cherry orchard (cultivars: Ferrovia, Lapins, Giorgia, Bigarreau Moreau, Staccato, Sweetheart) located 7 km west of the Gioia del Colle municipality in the Bari province (Figure 1). The orchard covered a total area of 7 hectares, and the cherry trees were approximately 12 to 15 years old at the time of the study. Parasitoids were released in the field as part of an experiment to assess their efficacy against *D. suzukii* populations.

The field was divided into two plots, 100 m from each other. *T. drosophilae* were released in the first plot, while the second plot served as an untreated control. The distance between the plots prevented parasitoid migration while ensuring similar environmental conditions in terms of climate, orientation, elevation, and plot size. The first release of *T. drosophilae* was carried out in August 2016 and involved 1000 adults in a ratio of 50:50 females and males. Other regular releases were carried out every week for about one year. The parasitoid population was monitored for 3 years after the first release.

*P. vindemiae*, instead, was naturally present in the experimental orchard. Although the primary aim of this study was to evaluate the field efficacy of *T. drosophilae*, the consistent presence and activity of *P. vindemiae* could not be ignored. This species was not part of the original experimental design; however, its consistent presence and unexpected abundance led us to include it in both field monitoring and laboratory assessments, as outlined in the following sections. The occurrence of *P. vindemiae* in Apulia is in line with previous reports from surveys across Italy [34]. Accordingly, the monitoring and laboratory operations include this second parasitoid as well, according to the procedure described hereafter.

#### 2.1.4. Monitoring of *T. drosophilae* Field Behavior

**Figure 2:**
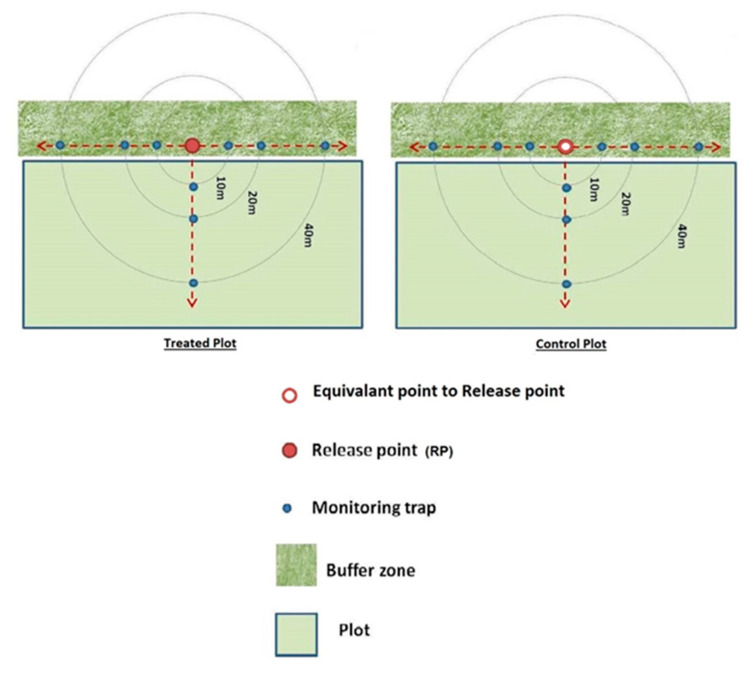
Scheme of experimental activities to assess the activity of the parasitoid under field conditions. This figure shows how traps were deployed into the treated and untreated parcels.

The distribution and parasitism rate of *T. drosophilae* under field conditions were assessed using traps placed within the two plots. In each plot, 9 traps were distributed between wild vegetation and crop trees, following 3 different directions. The traps were placed at 3 different distances (10 m, 20 m, and 40 m) from the parasitoid release point (treated field) or from an equivalent point in the control plot. This part of the study aimed to evaluate the potential parasitoid activity, its operating distance, and its ability to adapt in the field (Figure 2).

**Figure 3:**
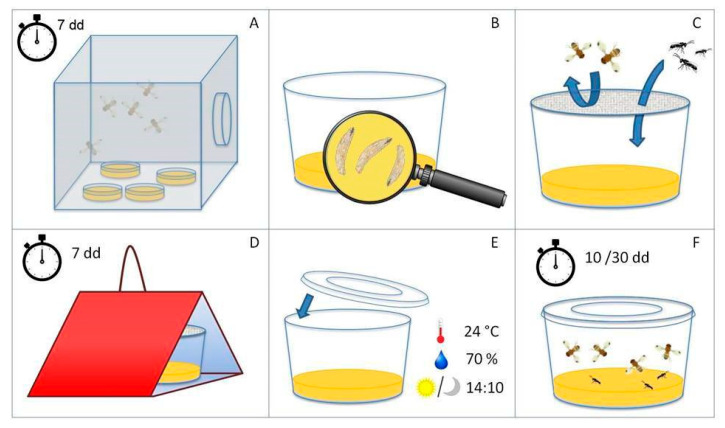
Monitoring of parasitoids on *Drosophila suzukii* artificially reared larvae. (**A**) Infestation of the artificial substrate by *D. suzukii* for 7 days. (**B**) Larval count (20 larvae/vail). (**C**) Fine mesh net covering the plastic cups. (**D**) Plastic cups were placed into the delta trap and exposed for 1 week into the field. (**E**) After 1 week, the plastic cups were collected from the field, sealed, and placed under controlled conditions. (**F**) The emergence of *D. suzukii* adults was counted for 10 days, while parasitoids adults were counted for 30 days.

The parasitism rate of the released parasitoids was also checked during the periods when cherries were not present in the orchard. Groups of 20 laboratory-reared third-instar larvae were isolated from the artificial substrate and moved into 350 mL vials containing a medium composed of 1–2 layers of thick fresh banana slices covered with a mixture of agar and water (20 mL of 7.05 g/L agar). Vials were covered with a fine-mesh net (20 mesh, 0.8 mm) that allowed for the entrance of parasitoids but not of drosophilids. Each vial was fixed inside a red delta trap (Figure 3), hung on trees 1.5–2 m from the ground. The vials were replaced weekly; the former ones were placed in growth chambers at 23 °C; 60–70% RH, 14 LD. The vials were checked 7 to 10 days after incubation to count the number of *D. suzukii* adults that had emerged; after counting, emerging specimens were removed to avoid double counts or mating and oviposition. The number of parasitoids that had emerged, instead, was counted 24–30 days after incubation (Figure 3).

Monitoring involved the *P. vindemiae* population as well: accordingly, after 24–30 days, the number of emerged parasitoids was recorded, distinguishing between *T. drosophilae* and *P. vindemiae*.

#### 2.1.5. Statistical Analysis

Statistical analyses were carried out using IBM SPSS Statistics 26 software. Prior to the analysis, the normality of the datasets was checked using the Shapiro test (α = 0.05). Differences in terms of *D. suzukii* specimens that emerged from the vials placed into the treated and control plots during the release period were analyzed using the Mann–Whitney U test, as the data were not normally distributed (*p* < 0.05). A Pearson correlation analysis was considered to evaluate the relationship between the abundance of *T. drosophilae* and the emergence of *D. suzukii* into the treated plot. Since these datasets were normally distributed (*p* > 0.05), a parametric correlation test was carried out.

The spread of *T. drosophilae*, instead, was analyzed as follows: the population abundance was correlated with distance from the release point using Pearson correlation analysis, as the datasets were normally distributed. Similarly, Pearson correlation analysis was carried out to explore the relationship between *D. suzukii* emergence and distance from the release point to evaluate whether suppression effects declined with distance. Additionally, to assess if *D. suzukii* emergence was significantly different between treated and control plots at specific distances (10 m, 20 m, 40 m), independent samples t-tests were carried out, as the data were normally distributed (*p* > 0.05). To analyze *T. drosophilae* abundance across different trap positions (right, left, center), we carried out a one-way ANOVA, as the abundance data followed a normal distribution across positions.

Differences in *P. vindemiae* abundance between the treated and control plots were explored through independent samples t-tests, as the data were normally distributed. The relationship between the abundance of *P. vindemiae* and the emergence of *D. suzukii* was assessed through Pearson correlation analysis in both plots. Additionally, to test if the strength of correlation was different between the treated and control plots, a Fisher’s Z-test was carried out.

Post-release period data were analyzed based on the Mann–Whitney U test, as the distribution of *T. drosophilae* abundance was not normal (*p* < 0.001). This analysis was carried out to check the population level of *T. drosophilae* in treated and control plots during the release and post-release periods to assess the orchard’s establishment.

To assess the potential effect of *T. drosophilae* release on *P. vindemiae* abundance, we carried out an independent sample *t*-test between the release (+), the first post-release (−), and the second post-release (−−) periods to assess the changes in abundance over time. In case the dataset was not normally distributed, we applied the Mann–Whitney U test.

The effect of the absence of *T. drosophilae* and the change in *P. vindemiae* abundance on *D. suzukii* emergence was assessed by analyzing the difference in the emergence between the release and post-release periods in the treated and untreated plots. This analysis was carried out through an independent sample t-test if data were normally distributed, and through the Mann–Whitney U test otherwise.

### 2.2. Interaction Between P. vindemiae and T. drosophilae Parasitism Rates on D. suzukii Under Laboratory Conditions

#### 2.2.1. Insect Rearing

*D. suzukii* adults were housed in ventilated Plexiglas cages located in a climatic chamber under controlled conditions of 22 ± 2 °C, 62 ± 4% RH, 12:12 h photoperiod, reared as explained in Section 2.1. *Trichopria drosophilae* and *P. vindemiae* were reared on third instar larvae and young pupae of *D. suzukii*. Eggs of *D. suzukii* were collected on the artificial diet and kept in growing chambers for 72 h to support the development of the third instar larvae and pupae. After the emergence of the target stage, host specimens were exposed to adult parasitoids for 48 h.

Three weeks after the exposition, adult parasitoids emerged from parasitized larvae, and pupae were collected, housed in separated cages, fed with a honey–sugar mixture, and supplied with water.

#### 2.2.2. Experimental Design

The interactions between *D. suzukii* and *T. drosophilae* and between *D. suzukii* and *P. vindemiae* were tested either independently or together. The experiment consisted of a no-choice assay containing 5 treatments combined with a free choices assay containing 2 treatments, for a total of 7 treatments. The trials were divided as follows.

No-choice assays:

T1 50 pupae of *D. suzukii* + 3 couples of *T. drosophilae*.
T2 50 pupae of *D. suzukii* + 3 couples of *P. vindemiae*.
T3 50 pupae of *D. suzukii* + 3 couples of *T. drosophilae* + 3 couples of *P. vindemiae*.
T4 50 pupae of *D. suzukii* left for 24 h with 3 couples of *T. drosophilae* + 3 couples of *P. vindemiae*.
T5 50 pupae of *D. suzukii* left for 24 h with 3 couples of *P. vindemiae* + 3 couples of *T. drosophilae*.

Free choice assays:

T6 50 pupae of *D. suzukii* left for 24 h with 3 couples of *P. vindemiae* + 50 pupae of *D. suzukii* + 3 couples of *T. drosophilae*
T7 50 pupae *D. suzukii* left for 24 h with 3 couples of *T. drosophilae* + 50 pupae *D. suzukii* + 3 couples of *P. vindemiae*.

Larvae of *D. suzukii* were collected, preserved in jars, and fed with artificial substrate (Section 2.1.1 *D. suzukii* rearing). When *D. suzukii* had reached the pupal stage, the previously mentioned 7 treatments were applied. The same protocol was repeated on grapefruits artificially exposed to *D. suzukii* adults for 48 h. After the exposition, the number of eggs laid was counted under a stereomicroscope. During the experiment, the number of *D. suzukii* eggs laid and adults emerged, the number of emerged parasitoids, and the number of non-emerged *D. suzukii* pupae were counted to compute the respective emergence rates of the three species as well as the parasitism rate of each parasitoid.

#### 2.2.3. Statistical Analysis

Statistical analyses of this second part of the study were carried out using IBM SPSS Statistics 26 software. In this case, the normal distribution of the datasets was checked prior to the analysis through the Shapiro test.

The effects of different parasitoid combinations on *D. suzukii* emergence was assessed through the Kruskal–Wallis test, as the dataset was not normally distributed. The test was followed by Bonferroni post hoc comparisons for multiple pairwise comparisons.

The Kruskal–Wallis test, followed by Bonferroni post hoc comparisons for multiple pairwise comparisons, was also applied to analyze the parasitism rates of *T. drosophilae* and *P. vindemiae* under different treatments and to detect significant differences among treatments.

## 3. Results

### 3.1. Effect of T. drosophilae and Its Interaction with P. vindemiae on D. suzukii Under Field Conditions

#### 3.1.1. Effect of *T. drosophilae* Releases on *D. suzukii* Suppression During the Release Period

**Figure 4:**
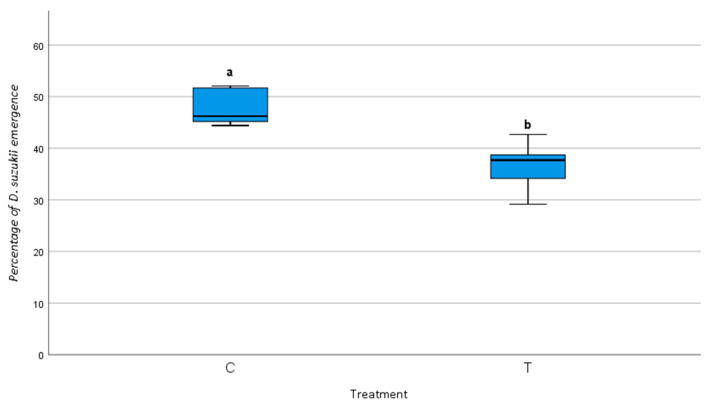
Difference in *Drosophila suzukii* emergence between the control (C) and treated (T) plots. Lines inside the boxes indicate median values, while the whiskers include 95% of the data. Different letters indicate statistical differences between the treatments assessed based on a Mann–Whitney U-test (α = 0.05).

The Mann–Whitney U test showed a significant difference in *D. suzukii* emergence between the treated and control plots just after the release of *T. drosophilae* (U = 0.000, Z = −3.578, *p* < 0.001), suggesting that the parasitoid significantly reduced *D. suzukii* emergence. The box plot in Figure 4 shows the differences in *D. suzukii* emergence between treated and control plots, highlighting the significantly lower emergence rates in the treated plot.

The regression analysis further indicated a significant negative correlation between *T. drosophilae* abundance and *D. suzukii* emergence (*r* = −0.670, *p* = 0.048), suggesting that *D. suzukii* emergence decreased as the abundance of *T. drosophilae* increased.

#### 3.1.2. Influence of Distance on *T. drosophilae* Dispersal and Effectiveness

The spatial distribution and movement of *T. drosophilae* after the release were assessed by analyzing the change in abundance with reference to trap distance (10 m, 20 m, and 40 m) from the release point in the treated plot. A significant negative correlation was observed between the abundance of *T. drosophilae* and the distance from the release point (r = −0.836, *p* = 0.005). Additionally, a significant positive correlation (r = 0.687, *p* = 0.041) between the distance and *D. suzukii* emergence indicates that *D. suzukii* emergence increased with increasing distance from the release point of *T. drosophilae*.

The effect of distance on *D. suzukii* population reduction was further analyzed by comparing the emergence levels in the treated and control plots at 10 m, 20 m, and 40 m. At 10 m, *D. suzukii* emergence was significantly lower (mean ± standard error) in the treated plot (6.3 ± 0.5 individuals) compared to the control plot (9.3 ± 0.2 individuals, t (4) = −9.136, *p* = 0.001), indicating a strong reduction effect within a short range. At 20 m, *D. suzukii* emergence was significantly lower in the treated plot (7.7 ± 0.8 individuals) than the control (9.9 ± 0.9 individuals, t (4) = −3.214, *p* = 0.032), suggesting that the influence of *T. drosophilae* may persist with a reduction at further distances. At 40 m, the differences in *D. suzukii* emergence between treated (7.9 ± 0.5 individuals) and control (9.4 ± 0.8 individuals) plots were marginally significant (t (4) = −2.721, *p* = 0.05), suggesting that control efficacy further decreased at this distance. Accordingly, *T. drosophilae* were more effective at 10 m, remained effective at 20 m, and weakened at 40 m from the release point.

The mean *T. drosophilae* abundance was highest in the central traps (1.6 ± 0.5 individuals), followed by the right (1.1 ± 0.5 individuals), and lowest was in the left traps (1.0 ± 0.5 individuals). However, these differences were not statistically significant, as indicated by the one-way ANOVA (F = 0.817, *p* = 0.485), suggesting that *T. drosophilae* distribution appeared uniform across the right, left, and center trap positions.

#### 3.1.3. Relationship Between *T. drosophilae* and *P. vindemiae* in *D. suzukii* Population Reduction

The abundance of *P. vindemiae*, which was naturally present in the orchard, was monitored in both treated plots (where *T. drosophilae* was released) and untreated control plots. No significant difference was found between the treated (Mean = 3.5 ± 0.8) and control plots (Mean = 3.0 ± 0.8) according to an independent samples *t*-test (t (16) = 1.03, *p* = 0.320), suggesting that the release of *T. drosophilae* did not negatively impact the abundance of *P. vindemiae*.

Further analysis highlighted a negative correlation between *P. vindemiae* abundance and *D. suzukii* emergence in the control plots (r = −0.965, *p* < 0.001), suggesting that *P. vindemiae* may provide a substantial contribution to controlling *D. suzukii* populations. In the treated plots, this correlation was weaker (r = −0.724, *p* = 0.027), indicating a possible interaction between the two parasitoid species. Fisher’s Z-test comparing correlation strength between the two conditions showed no statistically significant difference (Z = −1.748, *p* = 0.080), meaning that the presence of *T. drosophilae* did not significantly reduce the effectiveness of *P. vindemiae*.

#### 3.1.4. Interaction Between *T. drosophilae* and *P. vindemiae*

Multiple linear regression analysis showed a highly significant combined effect of *T. drosophilae* and *P. vindemiae* on *D. suzukii* emergence, F_2,6_ = 86.65, *p* < 0.001, accounting for 96.7% of the variability in *D. suzukii* emergence (R^2^ = 0.967). This result suggests that the joint action of the parasitoids significantly contributed to reducing the *D. suzukii* populations. In the treated plot, no significant correlation between *P. vindemiae* abundance and distance from the *T. drosophilae* release point was observed, meaning that the *P. vindemiae* populations did not increase as *T. drosophilae* abundance decreased with distance from the release point. Theoretically, this effect could have reduced competition for *D. suzukii* hosts.

#### 3.1.5. Post-Release Monitoring: Assessing *T. drosophilae* Adaptation and Its Long-Term Impact on *D. suzukii* and *P. vindemiae*

##### Parasitoid Presence in Treated and Control Plots and Persistence of *T. drosophilae* in the Field

During the releasing period (+), the data showed that both *T. drosophilae* and *P. vindemiae* were present in the treated plot, with *P. vindemiae* being more abundant. As expected, only *P. vindemiae* was present in the control plot. *T. drosophilae* specimens were no longer found in the treated or untreated plots during the post-release period; *P. vindemiae* remained present in both plots. According to the statistical analysis, *T. drosophilae* populations significantly decreased in the post-release (−) period (Mann–Whitney U = 0.000, *p* < 0.001), suggesting that the species did not establish itself in the field.

##### Changes in *P. vindemiae* Populations During the Post-Release Period

**Figure 5:**
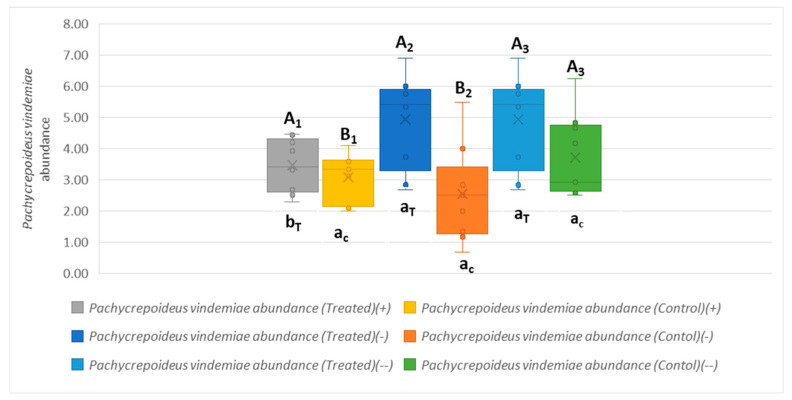
Variations in *Pachycrepoideus vindemiae* abundance across the release and post-release periods in the treated and control plots. Capital letters with subscripts (e.g., A_1_, B_1_) indicate statistical differences between the treated and control plots within the same period (_1_ = +, _2_ = −, _3_ = −−). Lowercase letters with subscripts (e.g., a_T_, b_T_, a_c_) indicate statistical differences between periods within the same treatment (T = treated, c = control). Lines and crosses within the boxes represent the median and the mean values, respectively.

*P. vindemiae* abundance was significantly higher in the treated plot in the first post-release year (−) (5 ± 1 individuals) (mean ± standard error) compared to the release period (+) (3.5 ± 0.8 individuals), t (16) = −2.571, *p* = 0.020. The Mann–Whitney U test showed no significant difference (U = 40.500, Z = 0.000, *p* = 1.000), with identical mean ranks mean rank (−) = 9.50, mean rank (−−) = 9.50) between the two post-release periods (− and −−) (Figure 5). This result shows that *P. vindemiae* populations increased in the first post-release year following the absence of *T. drosophilae* and remained stable thereafter, suggesting that this species compensated for the absence of *T. drosophilae* by becoming the dominant parasitoid in the treated plot, stabilizing its population in the field during the second post-release year.

In the control plot, the t-test showed that *P. vindemiae* abundance did not significantly differ between the release (+) and post-release (−) periods (*p* = 0.356), or between the first (−) and second (−−) post-release years (*p* = 0.095) (Figure 5). This result suggests that the observed increase in *P. vindemiae* abundance in the treatment plot might be the result of the lack of *T. drosophilae*, while *P. vindemiae* populations remained rather steady in the control plot.

**Figure 6:**
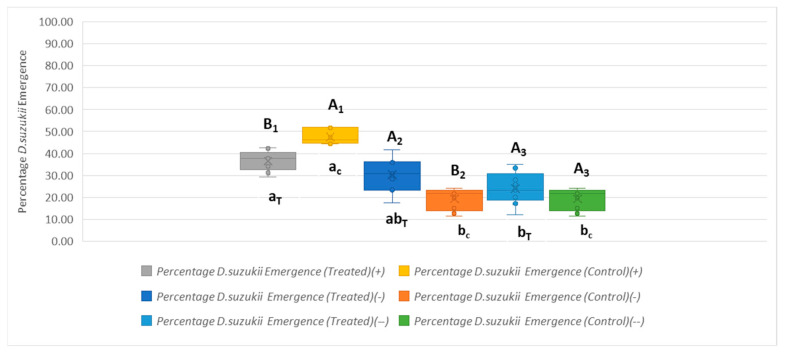
*Drosophila suzukii* emergence across the release and post-release periods in the treated and control plots. Capital letters with subscripts (e.g., A_1_, B_1_) indicate statistical differences between the treated and control plots within the same period (_1_ = +, _2_ = −, _3_ = −−). Lowercase letters with subscripts (e.g., a_T_, b_T_, a_c_, b_c_) indicate statistical differences between periods within the same treatment (T = treated, c = control).

Comparisons of the abundance of *P. vindemiae* in the treated and control plots during the first year after release showed a significantly higher abundance of *P. vindemiae* in the treated plot (5 ± 1 individuals) compared to the control plot (3 ± 1 individuals), t (16) = 3.394, *p* = 0.004 (Figure 6). The abundance of *P. vindemiae* in the treated and control plots during the second post-release year was not statistically different (Mann–Whitney, U = 22.000, Z = −1.634, *p* = 0.102).

##### Role of *P. vindemiae* in *D. suzukii* Population Reduction in the Absence of *T. drosophilae*

During the post-release period (−), *D. suzukii* emergence decreased significantly in the untreated plot (Mann–Whitney, mean rank (+) = 14.00, mean rank (−) = 5.00, U = 0.000, Z = −3.578, *p* < 0.001) (Figure 6). In the treated plot, the results showed a non-significant decrease in *D. suzukii* emergence between the release and post-release period ((+) = 7.3 ± 0.9, (−) = 6 ± 2), t (16) = 2.009, *p* = 0.062 (Figure 6). Moreover, *D. suzukii* emergence during the post-release period was significantly higher in the treatment plot (6 ± 2 individuals) compared to the control plot (4 ± 1), (t (16) = 3.593, *p* = 0.002) (Figure 6). These results suggest that, in the absence of *T. drosophilae*, *P. vindemiae* may have played a more prominent role in controlling *D. suzukii* in the untreated plot, while its effect in the treated plot was potentially diluted by prior ecological interactions with *T. drosophilae*.

Additionally, *D. suzukii*’s emergence between the first (−) and second (−−) post-release years showed no statistically significant difference in terms of *D. suzukii* emergence between the two periods (U = 40.500, Z = 0.000, *p* = 1.000) in the untreated plot, with identical mean ranks for both periods (mean rank (−) = 9.50, mean rank (−−) = 9.50) (Figure 6). In the treated plot, the results showed a non-significant decrease in *D. suzukii* emergence in the post-release period (6 ± 2 and 5 ± 1 individuals, t (16) = 1.782, *p* = 0.094) (Figure 6).

*Drosophila suzukii* emergence in the treated and control plots in the post-release (−−) period was not statistically significant (t (16) = 1.582, *p* = 0.133), with the mean emergence being higher in the treated plot (5 ± 1) compared to the control plot (4 ± 1) (Figure 6).

### 3.2. Effect of the Interaction Between P. vindemiae and T. drosophilae on the Control of D. suzukii and their Parasitism Rates Under Laboratory Conditions

#### *Drosophila suzukii* Emergence Across Different Parasitoid Combinations

The Kruskal–Wallis test followed by Bonferroni post hoc comparisons showed highly significant differences between treatments for both the artificial diet and grapes (*p* < 0.0001).

**Figure 7:**
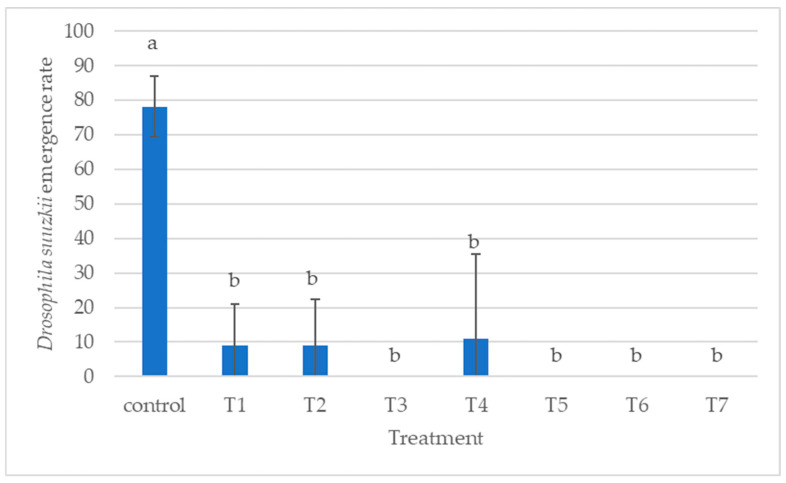
Effect of treatment on the emergence of *Drosophila suzukii* fed with an artificial diet (different letters indicate significant differences between treatments).

Figure 7 illustrates how different parasitoid treatments affected the emergence of *D. suzukii* when reared on an artificial diet. As expected, the control group, which received no parasitoids, showed the highest emergence rates. In contrast, most parasitoid treatments led to a strong reduction in fly emergence. In treatments T3, T5, T6, and T7, where both parasitoid species were applied together, no *D. suzukii* emerged. The only exception was T4, where *T. drosophilae* was introduced 24 h before *P. vindemiae*, and a small number of flies still emerged. However, the differences among the parasitoid treatments were not statistically significant.

**Figure 8:**
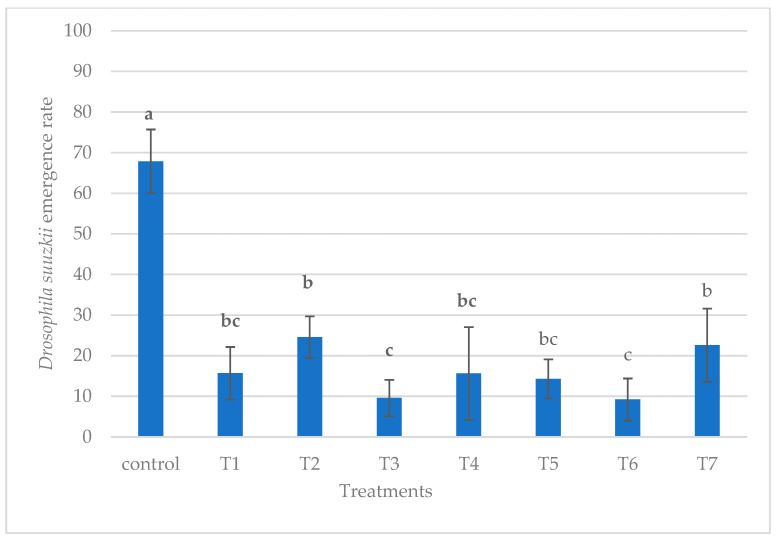
Effect of treatment on *Drosophila suzukii* emergence rate on grapes (different letters indicate significant differences between treatments).

Similar effects were observed when grapes were used as the substrate, in that the control group had the highest number of emerging *D. suzukii*. The lowest emergence rates were recorded when both *P. vindemiae* and *T. drosophilae* were introduced at the same time or sequentially (T3, T6) (Figure 8). It seems that the parasitism of a single parasitoid is enforced by the presence of an additional different parasitoid. *D. suzukii* emerged, suggesting that it is less effective by itself and works better when combined with *T. drosophilae*.

## 4. Discussion and Conclusions

This study evaluated the potential efficacy of *T. drosophilae* and *P. vindemiae*, two pupal parasitoids, in reducing *D. suzukii* infestations. Field trials showed that *T. drosophilae* had a stronger control effect during the release period. In the treated plot, where both parasitoids were present, *D. suzukii* emergence was significantly lower compared to the control plot. This outcome confirms the potential of *T. drosophilae* as a biological control agent and highlights the added value of combining both parasitoid species to enhance control efficacy.

The findings that the combined use of multiple parasitoids can increase parasitism rates and reduce *D. suzukii* more effectively than single-species release are in line with and add to those of previous studies [35]. The concurrent presence of both *T. drosophilae* and *P. vindemiae* in the treated plot led to higher levels of *D. suzukii* mortality, supporting the idea that species interactions may enhance biocontrol performance. Although coexistence with other species has proven to be an advantage, the effectiveness of *T. drosophilae* was spatially limited. This species was more abundant and effective near the release point, with its presence and impact decreasing significantly with distance, which suggests a limited dispersal capacity of *T. drosophilae*. These results are consistent with those reported in [30], in which it was found that pest reduction was most effective within 10 m from the release point. Moreover, the absence of significant differences in parasitism across left, center, and right trap positions suggests that the spread of the parasitoid was almost uniform in all directions from the release site.

These insights are valuable for optimizing future release strategies. To maximize efficacy, parasitoid release points should be spaced based on their dispersal capacity and the spatial configuration of the target area. The data presented herein highlights the importance of providing robust field evidence to arrange the resource allocation models predicted by precision agriculture approaches [36].

Despite the initial success, *T. drosophilae* was not detected in the treated plot after the release period, suggesting that it failed to become established. This failure could be due to unfavorable environmental conditions, insufficient host availability, or interspecific competition with *P. vindemiae*. For instance, *T. drosophilae* was reported to have reduced survival when coexisting with *P. vindemiae*, likely due to competition [37]. Similarly, dual parasitism of the same host led to decreased offspring production, further confirming the role of competition [38].

*P. vindemiae* may have held a competitive advantage over *T. drosophilae* due to its ectoparasitic strategy and dominant behavior in multi/parasitism scenarios. The low availability of hosts may have intensified this competition. Notably, after the disappearance of *T. drosophilae*, *P. vindemiae* abundance increased significantly in the treated plot during the first post-release year, while remaining stable in the control plot. This increase could reflect a compensatory response after the end of interspecific competition.

Nevertheless, the increase in *P. vindemiae* population was not sufficient to keep *D. suzukii* at the same low population level. In fact, *D. suzukii* emergence increased in the treated plot during the first post-release year, exceeding the levels observed in the control plot. Accordingly, the effectiveness of *P. vindemiae* in controlling *D. suzukii* may be lower than that of *T. drosophilae*. It is also possible that *P. vindemiae* can shift to alternative hosts in the presence of *T. drosophilae*, as suggested by Lee et al. [39], which may explain the low correlation between its abundance and *D. suzukii* population reduction.

The results obtained from laboratory trials supported the field observations. The emergence of *D. suzukii* was significantly higher under control conditions than in treatments with one or both parasitoids, either on an artificial diet or grapes. The combined presence of *T. drosophilae* and *P. vindemiae* led to the lowest emergence rates, indicating enhanced parasitism under competitive pressure. Accordingly, interspecific competition may lead to increased host exploitation, especially when space and host availability are limited.

The difference in parasitism potential between the tested parasitoids may be due to the biological traits of *P. vindemiae*, which, as an ectoparasitoid idiobiont, paralyzes the host externally before oviposition; in contrast, *T. drosophilae* is an endoparasitoid idiobiont that oviposits inside the host and later paralyzes it [35,40,41]. Even when *T. drosophilae* parasitizes a host first, *P. vindemiae* can still kill the host and produce offspring, showing its competitive strength [37]. In the opposite case, when *P. vindemiae* first parasitizes, *T. drosophilae* can still develop, but with a lower parasitism rate and fewer offspring, perhaps due to slower development and early resource competition. These factors can explain why *T. drosophilae* had trouble becoming established in the field.

Our results reinforce the idea that interspecific competition plays a key role in shaping the outcomes of biological control programs targeting *D. suzukii*. While *T. drosophilae* started more effectively, it likely lost ground over time due to competition. Interestingly, this idea contrasts with findings from da Costa Oliveira et al. [4], according to which another *Trichopria* species (*T. anastrephae*) outcompeted *P. vindemiae*. Therefore, competitive outcomes might depend on the specific traits of the parasitoid species involved. For instance, *T. anastrephae* is known to parasitize pupae inside fruit, making it more versatile [4]. In our case, *T. drosophilae* showed a more limited ability to disperse and exploit hosts, which might have led to its competitive disadvantage. Interestingly, our results showed that parasitoid species belonging to the same genus may overcome or be overcome in competition with the same competitor, likely because this divergence could be explained by biological differences between *T. anastrephae* and *T. drosophilae*, such as their ability to locate hosts in complex environments or their tolerance to interspecific competition. Taken together, our findings emphasize the importance of carefully selecting parasitoid species based on their ecological traits and competitive dynamics. Accordingly, the rationale behind this study can be helpful for further investigations that can extend current knowledge on new biocontrol agents, or for repeating the trials in geographical areas with different climate conditions.

In conclusion, although both *T. drosophilae* and *P. vindemiae* have proven to be promising biological control agents against *D. suzukii*, their use in combination may not be always straightforward. Their interaction may sometimes reduce overall parasitism due to interspecific competition. The success of such biocontrol strategies will likely depend on the timing and method of release. Sequential introductions or spatial separation could help minimize interference and better leverage the strengths of each species. Understanding these interactions is essential for designing more effective and sustainable biological control programs.

## Data Availability

The original contributions presented in this study are included in the article. Further inquiries can be directed to the corresponding author.
